# Infection spread simulation technology in a mixed state of multi variant viruses

**DOI:** 10.3934/publichealth.2022002

**Published:** 2021-11-05

**Authors:** Makoto Koizumi, Motoaki Utamura, Seiichi Kirikami

**Affiliations:** 1 PhD, Former researcher of Hitachi Ltd., Hitachi City, Ibaraki Prefecture, Japan; 2 PhD, PE, Former professor, Tokyo Institute of Technology, Tokyo, Japan; 3 Scholar, Former engineer of Hitachi Ltd., Hitachi City, Ibaraki Prefecture, Japan

**Keywords:** variant coronavirus, COVID-19, epidemiological model, prediction, multi-variant virus, delay differential equation

## Abstract

ATLM (Apparent Time Lag Model) was extended to simulate the spread of infection in a mixed state of the variant virus and original wild type. It is applied to the 4th wave of infection spread in Tokyo, and (1) the 4th wave bottoms out near the end of the state of emergency, and the number of infected people increases again. (2) The rate of increase will be mainly by d strain (L452R) virus, while the increase by a strain (N501Y) virus will be suppressed. (3) It is anticipated that the infection will spread during the Olympic Games. (4) When variant viruses compete, the infection of highly infectious virus rises sharply while the infection by weakly infectious ones has converged. (5) It is effective as an infection control measure to find an infected person early and shorten the period from infection to quarantine by PCR test or antigen test as a measure other than the vaccine.

## 1. Introduction

Mutation of SARS-CoV-2 has occurred in the infected areas of the United Kingdom, Brazil, and India over the past year. These variant viruses have also been brought to Japan. Variant viruses include those with weakened infectivity and those with increased infectivity. In the situation where several variant viruses coexist, it is considered that highly infectious virus trend to become the mainstream of the epidemic. In order to suppress the infection spreads, it is necessary to take measures depending on the virus. In constructing infection control measures, it is necessary to predict whether the infection will spread or shrink. To predict the infection spread, several calculation models were proposed.

First, we take a look at the prediction methods that have been used to date. SIR [1] and SEIR [2] models are often used in early infection stage. Because, they are simple mathematical structure and require short calculation time. As the progressing of infection spreads, measures such as vaccination and lockdown will be taken. Kuniya et al. use SEIR to evaluate the effect of the SOE (State Of Emergency) in the second wave in Tokyo. They conducted a parameter survey with varying coefficients of the equation [3]. Britton et al. applied improved SEIR to spread infection under the case of non-uniform population structure [4]. Muñoz-Fernández et al. applied the modified SIR model to analyze the wave of COVID-19. They used nonconstant parameter [5]. Biala et al. improved SEIR model to calculate the spread of COVID-19 pandemic [6]. On the other hand, ABM (Agent Based Model) has been developed [7]–[11]. This technology is a probabilistic method, and unlike the deterministic methods such as SIR and SEIR, it is a method that assumes various behaviors of a person and calculates the infection probability, and it takes a considerable amount of calculation time. Many calculation methods as mentioned above are constructed assuming a single virus, and do not consider the mixture of variant viruses. The objective of our research is to develop a technology for predicting the spread of infection in a mixed state of variant viruses.

Next, we briefly describe the progress of research so far. The above methods do not have a time delay from infection to quarantine. We considered that the time required until isolated from infected is the important role of contribution in expanding infection, therefore we developed ATLM (Apparent Time Lag Model) with a delay until isolation time [12]. This model currently has an extended version with vaccine and lockdown effects [13]. We have expanded it to handle variant viruses. The infectivity of variant viruses has already been reported [14]. We use these data to simulate the fourth wave of infection spread in Tokyo and investigate the availability of the method.

## 2. Method

### 2.1. Analysis model

The ATLM we have developed [12] uses the following equation, which takes into account the time delay from infection to quarantine and the time delay from infection to loss of infectivity. We denote the cumulative number of infected people by *x* as unknown, and daily infected people is by *dx*/*dt*.

$$\frac{dx}{dt}=\alpha \left(t\right)\left\{x\left(t\right)-\left(1-\varepsilon \right)x\left(t-T\right)-\varepsilon x\left(t-S\right)\right\}\left(1-\frac{x\left(t\right)}{M\left(t\right)}\right)$$ (1)

$$\alpha (t)=\alpha_{0}\rho \left(t\right)$$ (2)

$$M(t)=M_{0}\left(1-µ(t)\right)$$ (3)

where, *T*: time delay from infection to quarantine, *µ*(*t*): vaccination rate, α: infectivity, *ε*: ratio of asymptomatic persons, *S* is time delay from infection to the extinction of infectivity. *M* indicates the sensitive population. *ρ*(*t*) is the rate of decrease in infectivity due to the restriction of human flow such as a lockdown. α_0_ is original infectivity of the virus. Equation (3) represents the decrease in the sensitive population due to vaccination. Subscript 0 indicate initial value. Details of these equations were shown in previous paper [12].

Number of quarantined persons in *y*(*t*) and number of infected people during infection isolation in a community *z*(*t*) can be calculated by Eqs. (4) and (5) respectively.

y(t)=(1−ε)(x(t−T)−x(t−S)) (4)

z(t)=x(t)−(1−ε)x(t−T) (5)

To extend the above equations for handling variant viruses, the following assumptions are taken into account.

(1) Infected people are infected with only one type of virus, and there is no simultaneous infection.

(2) Patients who have been infected with one variant virus in the past are not infected with another variant virus.

(3) The infection rates between viruses are independent of each other and do not interfere with each other.

(4) The effect of the vaccine is the same for each variant virus.

(5) Both delay times until the onset and the infectivity disappear are the same for each variant virus.

Under the above assumptions, cumulative number of people infected by variant virus *i* is expressed by subscript *i*. the differential Eq. (1) is rewritten as follows.

$$\frac{dx_{i}}{dt}=\alpha_{i}\left(t\right)\{x_{i}\left(t\right)-\left(1-\varepsilon \right)x_{i}\left(t-T\right)-\varepsilon x_{i}\left(t-S\right)\}\left(1-\frac{X\left(t\right)}{M(t)}\right)$$ (6)

$$X\left(t\right)=\underset{i}{\sum }x_{i}\left(t\right)$$ (7)

where *a_i_* is infectivity of variant virus *i*. Equation (7) is a limitation induced from assumptions (1) and (2). That is, it is shown that variant viruses have the common sensitive population *M*. Number of quarantined persons in each virus *y_i_*(*t*) and number of infected people during infection isolation in a community *z_i_*(*t*) are also able to be calculated by following equations.

$$y_{i}\left(t\right)=\left(1-\varepsilon \right)\left(x_{i}\left(t-T\right)-x_{i}\left(t-S\right)\right)$$ (8)

$$z_{i}\left(t\right)=x_{i}\left(t\right)-\left(1-\varepsilon \right)x_{i}\left(t-T\right)$$ (9)

### 2.2. Time integration

The analytical solution of the differential Eq. (4) is unknown. Therefore, to solve the above equations, the 4th-order Runge-Kutta method was used for numerical integration. In Eq. (4), the numerical values of *x_i_*(*t-T* ) and *x_i_*(*t-S*) at time *t* have been already computed and there is no problem in accuracy. However, since these values are not calculated at the start of the calculation, precaution must be taken at the start time. Therefore, the initial value is given a sufficiently small value compared to *M*_0_. Next, it should be noted that, when *X*(*t*) is increased, too close to *M*(*t*). Especially when the vaccination rate becomes high, *X(t)* > *M(t)* may occur, in which case the solution oscillates and becomes unstable. To avoid numerical unstable, if *X(t)*/*M(t)* > 1, we set the right-hand side of Eq. (4) equal zero.

## 3. Application to the 4th wave of infection spread in Tokyo

### 3.1. Analysis conditions

**Table 1.** Calculation conditions.

	α strain (N501Y)	δ strain (L452R)
Initial Infected People	4400	5.5
Infectivity	0.09	0.121
Sensitive Population	500000
Start Point	2021/3/1
Vaccination Start	2021/5/15
Vaccination Rate	0.42%/day
Start of SOE	2021/4/25
End of SOE	2021/6/20
Time Delay until Quarantine	14 days
Quarantine Period	14 days
Ratio of Asymptomatic Persons	0

Note: SOE: State Of Emergency.

Variant virus that is prevalent in Tokyo and is seen as almost by a strain virus (N501Y) [14]. Currently, the virus of interest is d strain (L452R) found in India. Infectivity of a strain prevalent in Tokyo is believed as 1.32 times that of the original wild type [14]. Infectivity of d strain is estimated at 1.78 times higher than original ones [15]. Thus, the ratio between two becomes about 1.35. We set the infectivity of each virus based on this ratio. It is confirmed that 9 people are infected at 2021/5/31 by d strain. Its sampling percentage of infected people is 10%, therefore, about 90 people are infected [15]. Table 1 shows calculation conditions. The initial value by d strain was determined so as to satisfy the above conditions. See appendix A. As shown in the table, the effect of the vaccine is incorporated. The SOE (State Of Emergency) was scheduled by Japanese Government and Tokyo Metropolitan Government.

### 3.2. Analysis results

**Figure 1.**
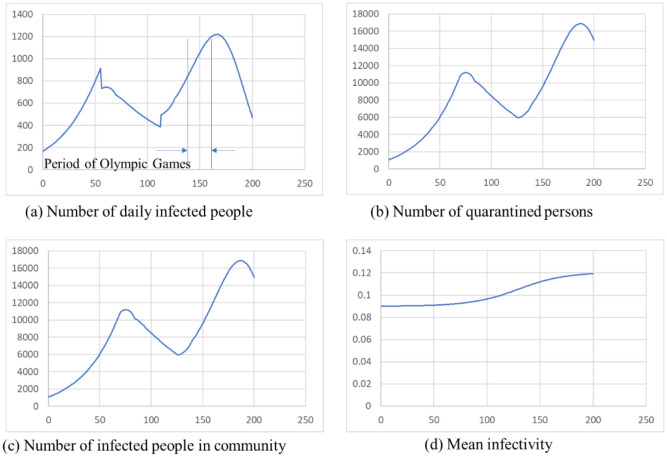
The pattern of 4th wave in Tokyo.

Figure 1 shows the pattern of the 4th wave in Tokyo. The daily change in the number of infected people is illustrated in (a). The origin of the horizontal axis is set at 2021/3/1. The epidemic peak is located at around fifty days from the calculation start (the beginning of May or the end of April), about 800 people infected persons have been calculated. This number is roughly equivalent to the actual 7-day average for the 4th wave. (b) shows the number of quarantined persons calculated by Eq. (6), including home medical treatment and hotel medical treatment. It is said that 80% of the infected persons are mild according to the WHO. Hence, we estimated that remaining 20% would be in the hospital. Therefore, at the peak more 11,000 people has been quarantined and about 2200 people is considered in the hospital. According to the data of Tokyo [15] at the time of the fourth wave peak about 2400 patients were hospitalized, then the results are consistent with the actual data. (c) displays number of infected people until isolated in a community calculated by Eq. (9). The higher this number, the higher the probability of having the next infected person. The average infectivity of the variant virus is shown in (d), and as the infection progresses, the average value becomes closer to the infectivity of strain. It shows that the strain is becoming dominant.

**Figure 2.**
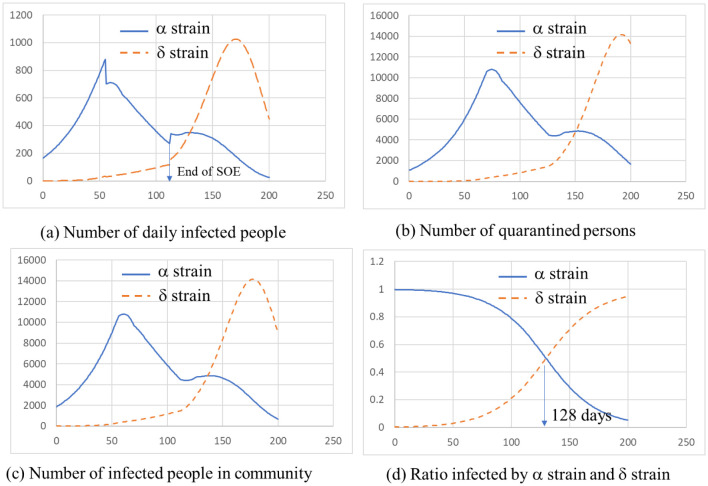
Changing number of infected people by each virus.

The changing number of infected people by each virus are displayed in Figure 2. (a), (b) and (c) show the number of daily infected persons, quarantined persons, and infected persons in the community respectively. Solid line is the number of people infected by a, and broken line is due to d. Each figure shows that the patients by d has increased sharply after the end of the SOE. (d) in Figure 2 shows a ratio of patients by a and d. The patients by d increases from the SOE declared the end and becomes dominant after the point of 128 days (2021/7/6) from the calculation start. In addition, from these figures, it can be seen that the infection by d rises sharply at the stage when the infection of a has converged and bottomed out. As described above, this analysis also indicated that the one with stronger infectivity became dominant when the infection spreads.

## 4. Discussion

### 4.1. Sensitivity analysis

**Figure 3.**
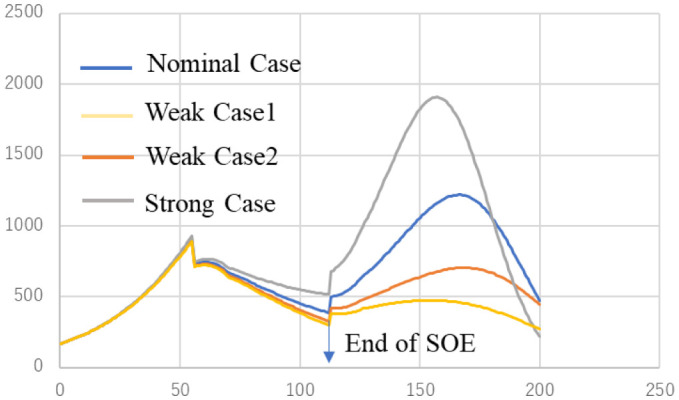
Change of infected people due to differences in infectivity.

We have already examined the sensitivity about time delay *T* in the previous work [12] and reproductive number *R* is proportion to product of infectivity *α* and time delay *T*. Therefor less *T* gives suppress of spread of infection. The effect of vaccination was also considered in the recent paper [13]. Then, in this section, we examine the effect of infectivity difference under the coexistence of two variant viruses. Calculation conditions are shown in Table 2. Infectivity of α strain is set to constant and that of δ strain is changed from −8.2% to 4.1%. The results are displayed in Figure 3. In strong case, maximum infected people after SOE becomes about 1800, on the other hand, in weak case1 and weak case 2, maximum values become about 500 and 700 respectively. These results suggest that the ratio of infectivity between δ strain and α strain over 1.3 accelerates replacement of α by δ.

**Table 2.** Infectivity for sensitive analysis.

	α strain	δ strain	Difference (%)	Ratio (δ/α)
Nominal Case	0.09	0.121	0	1.35
Weak Case 1	0.09	0.111	−8.2	1.23
Weak Case 2	0.09	0.116	−4.1	1.29
Strong Case	0.09	0.126	4.1	1.4

The problems of reinfection and breakthrough infection will be more important. To solve these problems, rate of decrease in antibody level or probability of reinfection and breakthrough infection must be taken into account. In the present calculation model has not yet adopted the methodology to solve above problems. Improvement of the model is future task.

### 4.2. Measures to suppress spread of infection

The above calculation results predict the peak of infection will become during the Olympic Games in Tokyo. Then, we thought three measures as follows.

(1) In the case of continuing of the current measures (Case 1).

(2) Measure 1: In the case of extending the SOE to 6/30 (Case 2).

(3) Measure 2: To shorten the period from infection to isolation by PCR test or antigen test (Case 3).

**Figure 4.**
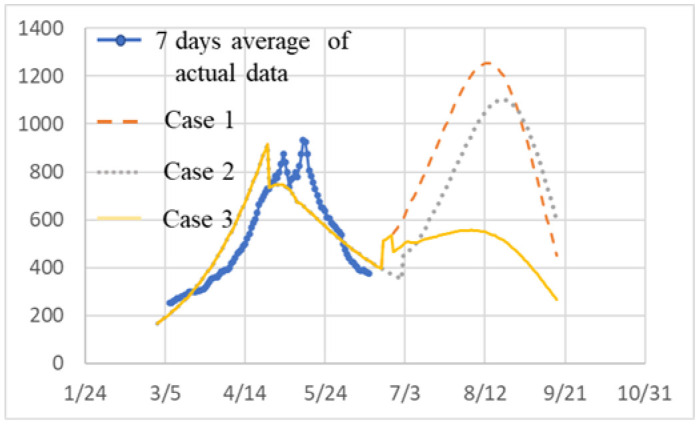
Infection status in case.

The infection status of each case is plotted together in Figure 4. The horizontal axis is the date from 2021/3/1. The broken line, dotted line and solid line indicate Case 1, Case 2, and Case 3 respectively. Solid line with blue dots shows the infection status in Tokyo on a 7-day average [15].

Case 2 shows the transition of infected persons when the SOE is extended to 6/30. Peak of infected persons decreases by about 100, however the big improvement of the infection situation is not observed. Therefore, the extension until 6/30 has little effect on suppressing infection.

The last measure is not effective unless as many people as possible participate. It is good if you know that you will be infected, but usually you do not know, so you need to have many people check it regularly. For that purpose, a negative certificate with a time limit (up to one week) should be issued and confirmed at restaurants and event venues. This will allow many people to be tested. In this way, it is possible to shorten the period from infection to isolation for two days or one day. In the present study, this period is set to 14 days due to the consistency of the data. We set the period to 12 days. Considering five days as a preparation period after the end of SOE, the implementation date was set to 6/25. Case 3 shows that the spread of infection after the peak of the 4th wave is suppressed to about 600. Searching for infected people early and shortening the infection period in this way is the most effective method other than vaccines as an infection control measure.

## 5. Conclusions

We have extended the ATLM which has been developed to simulate the status of infection with various variant viruses. The developed model was applied to the 4th wave of Tokyo and the following results were obtained.

(1) The fourth wave will bottom out near the end of the state of emergency, and the number of infected people will increase again.

(2) The rate of increase will be mainly by δ strain, while the increase in α strain will be suppressed.

(3) It is anticipated that the infection will spread during the Olympic Games.

(4) When variant viruses compete, the infection of strongly infectious one rises sharply while the infection by weak infectious ones has converged.

(5) The results of sensitivity analysis suggest that the ratio of infectivity between δ strain and α strain over 1.3 accelerates replacing speed of dominant virus in infection spread.

(6) It is effective as an infection control measure to find an infected person early and shorten the period from infection to quarantine by PCR test or antigen test as a measure other than the vaccine.
