# Supplementary material for: Infection spread simulation technology in a mixed state of multi variant viruses
Source: AIMS Public Health. 2021 Nov 5;9(1):17–25. doi: 10.3934/publichealth.2022002 (PMC8755968; doi:10.3934/publichealth.2022002)
Supplement: Supplementary file 1 [file publichealth-09-01-002-s001.pdf]

Public Health

AIMS Public Health, 9(1): 17–25.
DOI: 10.3934/publichealth.2022002
Received: 16 August 2021
Accepted: 01 November 2021
Published: 05 November 2021

http://www.aimspress.com/journal/aimsph

Research article

Infection spread simulation technology in a mixed state of multi variant viruses

Makoto Koizumi[1,*], Motoaki Utamura[2] and Seiichi Kirikami[3]

[1] PhD, Former researcher of Hitachi Ltd., Hitachi City, Ibaraki Prefecture, Japan
[2] PhD, PE, Former professor, Tokyo Institute of Technology, Tokyo, Japan
[3] Scholar, Former engineer of Hitachi Ltd., Hitachi City, Ibaraki Prefecture, Japan

* **Correspondence:** Email: koizumi@theia.con.ne.jp.

Appendix A

Calculation of the first approximation of M and α

In ATLM, the rate of infections (daily new cases) $\frac{dx(t)}{dt}$ is given by the following equation with the collective population M and the transmission rate α.

$$\frac{dx(t)}{dt} = \alpha\{x(t) - x(t-T)u(t-T)\}\left(1 - \frac{x(t)}{M}\right) \qquad (A-1)$$

here, $x(t)$: cumulative infections, T: delay time from infection to quarantine, u: the step function.

Since daily new cases varies significantly, integration is used to smooth them.

The intervals $[t_1, t_2], [t_3, t_4] (0 < t_1 < t_2 < t_3 < t_4)$ are used as those of interest.

The following equation is obtained from the integration of (A-1) in the interval $[t_1, t_2]$.

$$\int_{t_1}^{t_2} \frac{dx(t)}{dt} dt = \alpha\{\int_{t_1}^{t_2} \big(x(t) - x(t-T)\big) dt - \frac{\int_{t_1}^{t_2} \big(x(t) - x(t-T)\big) x(t) dt}{M}\} \qquad (A-2)$$

Normally, $T < t_1$. For this reason, we set the step function u = 1.
(A-2) is modified to obtain (A-3).

$$\frac{\int_{t_1}^{t_2}\big(x(t)-x(t-T)\big)x(t)dt}{M\int_{t_1}^{t_2}\big(x(t)-x(t-T)\big)dt}+\frac{\int_{t_1}^{t_2}\frac{dx(t)}{dt}dt}{\alpha\int_{t_1}^{t_2}(x(t)-x(t-T))dt}=1 \qquad (\mathrm{A}-3)$$

Similarly, (A-4) is obtained in the interval$[t_3, t_4]$.

$$\frac{\int_{t_3}^{t_4}\big(x(t)-x(t-T)\big)x(t)dt}{M\int_{t_3}^{t_4}\big(x(t)-x(t-T)\big)dt}+\frac{\int_{t_3}^{t_4}\frac{dx(t)}{dt}dt}{\alpha\int_{t_3}^{t_4}(x(t)-x(t-T))dt}=1 \qquad (\mathrm{A}-4)$$

(A-3) and (A-4) are linear equations of $\frac{1}{M}$and$\frac{1}{\alpha}$. Then M and α are calculated.

Usually, the following inequality holds in the range of monotonously increasing$\frac{dx(t)}{dt}$.

$$\frac{\int_{t_1}^{t_2}\big(x(t)-x(t-T)\big)x(t)dt}{\int_{t_1}^{t_2}\big(x(t)-x(t-T)\big)dt}<\frac{\int_{t_3}^{t_4}(x(t)-x(t-T))x(t)dt}{\int_{t_3}^{t_4}(x(t)-x(t-T))dt}$$

The condition (A-5) is sufficient for M and α to be positive.

$$\frac{\int_{t_1}^{t_2}\big(x(t)-x(t-T)\big)dt}{\int_{t_1}^{t_2}\frac{dx(t)}{dt}dt}<\frac{\int_{t_3}^{t_4}(x(t)-x(t-T))dt}{\int_{t_3}^{t_4}\frac{dx(t)}{dt}dt} \qquad (\mathrm{A}-5)$$

The case of Tokyo.

The source is from the following.

Updates on COVID-19 in Tokyo. Available from: https://stopcovid19.metro.tokyo.lg.jp/en/.

The intervals were 2021 3/22–4/4 (14 days) and 4/5–4/18 (14 days) before the state of emergency. The delay time from infection to quarantine was T = 14 days. Therefore, the measured number of daily new cases$\frac{dx(t)}{dt}$on the day *t* was used as the number of daily new ones T = 14 days ago.

The cumulative infections $x(t)$ were accumulated from the minimum number of daily new cases on February 15th.

$$\text{Setting } \frac{1}{M} -> x, \frac{1}{\alpha} -> y,$$

we obtained the following two linear equations.

$$\frac{x}{0.00004129}+\frac{y}{11.729}=1$$

$$\frac{x}{0.00006737} + \frac{y}{11.416} = 1$$

from these equations, M = 3.66013*10^5 and α = 0.0913 were obtained as the first approximations.

Integration was calculated with the trapezoidal formula. It was also used for the number of daily new cases$\frac{dx(t)}{dt}$. Therefore, numerically,

$$\int_{t_1}^{t_2} \frac{dx(t)}{dt} dt \neq x(t_2) - x(t_1)$$

In the above, the calculation of the first approximations of M and α is shown by taking the case of Tokyo. These must be so tuned that they are consistent with the other measurements.

AIMS Press

© 2022 the Author(s), licensee AIMS Press. This is an open access article distributed under the terms of the Creative Commons Attribution License (http://creativecommons.org/licenses/by/4.0)
